# MicroRNA-22-3p and MicroRNA-149-5p Inhibit Human Hepatocellular Carcinoma Cell Growth and Metastasis Properties by Regulating Methylenetetrahydrofolate Reductase

**DOI:** 10.3390/cimb44020063

**Published:** 2022-02-16

**Authors:** Chao Li, Xiang Li, Han Wang, Xihan Guo, Jinglun Xue, Xu Wang, Juan Ni

**Affiliations:** 1 School of Life Sciences, The Engineering Research Center of Sustainable Development and Utilization of Biomass Energy, Yunnan Normal University, Kunming 650500, China; lichao@fudan.edu.cn (C.L.); lixiang7368@163.com (X.L.); wanghan9215@163.com (H.W.); guoxh1987@163.com (X.G.); 2 State Key Laboratory of Genetic Engineering, Institute of Genetics, School of Life Sciences, Fudan University, Shanghai 200438, China; jlxue@fudan.edu.cn

**Keywords:** microRNAs, hepatocellular carcinoma, methylenetetrahydrofolate reductase, growth, metastasis

## Abstract

microRNAs are small endogenous noncoding RNAs that have emerged as key negative regulators that target gene expression through RISC. Our previous study showed that the methylenetetrahydrofolate reductase gene (*MTHFR)* plays a key role in one carbon metabolism, which is downregulated by miR-22-3p and miR-149-5p, and that it could exert a potential anti-cancer effect. Whether miR-22-3p/miR-149-5p can regulate MTHFR to exert anti-cancer effects has become the focus of our research. Normal (HL-7702 cells) and cancerous (QGY-7703/HepG2 cells) human hepatocellular cells were transfected with 100 nM hsa-miR-22-3p/hsa-miR-149-5p mimic or controls. After 24, 48, and 72 h, cell proliferation ability was tested using CCK-8. The changes in *MTHFR* expression at both the transcriptional and translational levels were determined by RT-qPCR and Western blotting, respectively. Cancerous cell invasion and migration ability were confirmed by means of a transwell assay. We found that ectopic miR-22-3p/miR-149-5p inhibits hepatocellular carcinoma cell proliferation but does not inhibit normal human hepatocyte proliferation. The transfection of ectopic miR-22-3p/miR-149-5p downregulated the *MTHFR* expression in QGY-7703 and HepG2 but not in HL-7702. QGY-7703 and HepG2 migration and invasion were inhibited by ectopic miR-22-3p/miR-149-5p. Additionally, we found that ectopic miR-22-3p/miR-149-5p significantly increased the expression of *TP53INP1* and *PDCD4* in QGY-7703. The results of the study suggest that miRNA-22-3p and miRNA-149-5p inhibit tumor growth and metastasis properties may be by regulating MTHFR and that they exert anticancer effects in hepatocellular carcinoma cells.

## 1. Introduction

Hepatocellular carcinoma (HCC) is the fifth most common cancer worldwide and the third most frequent cause of cancer-related death [1]. The degree of malignancy of hepatocellular carcinoma is high, the disease progresses rapidly, and the survival time of the patient is relatively short. In recent years, hepatocellular carcinoma treatments have developed rapidly, and these treatment methods are diverse and include surgical treatment (hepatectomy, lobectomy, and liver transplantation), physiotherapy (microwave, radiofrequency ablation), chemotherapy (chemotherapy pump chemotherapy and intervention chemotherapy), and the recently developed of molecular targeted therapy method (bio-missiles); however, the overall survival rate of liver cancer patients is still relatively low among cancer patients.

MicroRNAs (miRNAs) are small endogenous noncoding RNAs (19–24 nucleotides in length) that have emerged as key negative regulators that target gene expression at the post-transcriptional level [2,3]. In humans, 2578 mature miRNA sequences are predicted to exist [4]. MicroRNAs have multiple targets, and several miRNAs can also meticulously regulate the same gene simultaneously. MiRNAs contribute to various aspects of animal development and/or human disease [5,6,7].

*Methylenetetrahydrofolate reductase* (*MTHFR*) serves as a key shift gene between DNA methylation and synthesis in one-carbon metabolism (OCM, or folate metabolism) through the conversion of 5, 10-methyltetrahydrofolate to 5-methyltetrahydrofolate (5-MTHF). The compound 5-MTHF lends a methyl group to homocysteine to generate methionine, which participates in methylation. In contrast, the loss of a methyl group from 5-MTHF results in the generation of tetrahydrofolate, which participates in thymine synthesis. Abnormal *MTHFR* function is related to an imbalance of DNA synthesis, methylation, and cellular replication as well as low folate status in the plasma/red blood cells, resulting in the accumulation of plasma homocysteine, further increasing oxidative stress (*P53* oxidative lesions)/endothelial dysfunction. Reduced *MTHFR* activity is associated with a high-risk for the development of HCC and is correlated with a lower risk of late-stage HCC and favorable survival outcomes in patients with HCC [8,9,10].

Our previous study showed that *MTHFR* is downregulated by miR-22-3p and miR-149-5p, which may have a potential anti-cancer effect [11]. This study aimed to verify and clarify the anti-cancer effect of miR-22-3p/miR-149-5p.

## 2. Methods

### 2.1. Cell Culture

The normal human hepatocyte cell line HL-7702 (cat# GNHu 6, Cell Bank of the Chinese Academy of Sciences) and human hepatocellular carcinoma cell line QGY-7703 (cat# TCHu 43, Cell Bank of the Chinese Academy of Sciences) were cultured in RPMI-1640 medium (GIBCO, Grand Island, NY, USA). The human hepatocellular carcinoma cell line HepG2 (cat# HB-8065, ATCC) was cultured in Dulbecco’s modified Eagle medium (DMEM) (GIBCO, Grand Island, NY, USA). Both media were supplemented with 10% fetal bovine serum (GIBCO, Grand Island, NY, USA) and 0.1% penicillin/streptomycin (GIBCO, Grand Island, NY, USA). L-Glutamine (1%; GIBCO, Grand Island, NY, USA) was added to the culture medium immediately before use. All cultures were maintained at 37 °C in a humidified atmosphere with 5% CO_2_.

### 2.2. MiRNA Mimic Transfection

HL-7702, QGY-7703, and HepG2 were plated in 12- or 96-well (for CCK-8 cell proliferation assay) culture plates (Costar, Corning, Inc., New York, NY, USA) at a density of 1 × 10^6^ cells or 2000 cells (96-well) per well. Lipofectamine 2000 (Invitrogen, Carlsbad, CA, USA) was used to transfect the cells with hsa-miR-22-3p/hsa-miR-149-5p mimic or controls (100 nM, RIBOBIO, Guangzhou, China). A total of 24, 48, or 72 h after transfection, the cells were washed three times in PBS, and the total RNA, miRNA, and protein were extracted separately.

### 2.3. CCK-8 Detects Cell Proliferation

Cell suspensions (2000 cells/100 μL/well) were prepared by transfecting a certain amount of HL-7702, QGY-7703, and HepG2 cells in a 96-well plate, and the plate was placed in a 37 °C, 5% CO_2_ incubator. Additionally, 10 μL of CCK-8 solution was added to each well (and attention was paid so as to not create air bubbles in the well, which would have affected OD readings). The plates were incubated in the incubator for 1 to 4 h. The absorbance at 450 nm was measured using a multi-plate reader.

### 2.4. Real-Time miRNA/mRNA Expression Analyses

MiRNAs were purified by using an miRNA Isolation Kit (Omega, Norcross, GA, USA). Polyadenylation and reverse transcription were performed by using the miRcute Plus miRNA first-strand cDNA Synthesis Kit (TIANGEN). For the poly (A) tailing RT-qPCR analyses, we used the miRcute miRNA qPCR Detection Kit (SYBR Green). The miRNA primers that were used in our study were purchased from the TIANGEN Human miRNA Specific qPCR Primer Sets (TIANGEN). The PCR reaction was performed in an ABI Step One Plus PCR System (Applied Biosystems, Foster City, CA, USA).

Total RNA was isolated from cells with the miRNA Isolation Kit (Omega, USA), and the quality of the RNA was assessed using a Nano Photometer (IMPLEN, München, Germany). The RNA samples that were obtained at 1 μg/μL were used for the reverse transcription reaction (Reverse Transcriptase Kit, Takara, Dalian, China). For the quantitative real-time qPCR (RT-qPCR) analyses, we used an SYBR RT-PCR Kit (KAPA, Wilmington, MA, USA). The *MTHFR* primers were: 5′-CACTACGGTGGGCTGGA TGA-3′ (forward) and 5′-GCTCCGGGTTAATTACCACCTTG-3′ (reverse). The programmed cell death protein 4 (*PDCD4*) primers were: 5′-ATGAGCACAACTGATGTGGA AA-3′ (forward) and 5′-ACAGCTCTAGCAATAAACTGGC-3′ (reverse). The tumor protein p53-inducible nuclear protein 1 (*TP53INP1*) primers were: 5′-GCACCCTTC AGTCTTTTCCTGTT-3′ (forward) and 5′-GGAGAAAGCAGGAATCACTTGTAT-3′ (reverse). The GAPDH primers were: 5′-GGCACAGTCAAGGCTGAGAATG-3′ (forward) and 5′-ATGGTGGTGAAGACGCCAGTA-3′ (reverse). The relative expression level of each gene was normalized to that of *GAPDH* using the 2^−ΔΔCt^ cycle threshold method.

### 2.5. Western Blotting Analyses

The cells were disrupted in cell lysis buffer (cat# P0013, Beyotime, Shanghai, China) supplemented with PMSF (Biosharp, Shanghai, China) and centrifuged for 15 min at 4 °C. The protein concentrations were determined using a BCA protein assay (Beyotime, Shanghai, China). Then, 30 μg of protein was resolved by SDS-PAGE using 10% Tris-glycine gels. After SDS-PAGE, the separated proteins were transferred to polyvinylidene fluoride (PVDF) membranes (Millipore, Burlington, MA, USA) and blocked for 2 h at room temperature with 5% skim milk/TBST (Tris-Buffered Saline with Tween-20). After incubation with an anti-human *MTHFR* (1:2000; Abcam, Cambridge, MA, USA) primary antibody under blocking conditions, the proteins were detected using an anti-mouse HRP-conjugated secondary antibody (1:10,000; Vazyme, Nanjing, China) and enhanced chemiluminescence (ECL) (Millipore, Burlington, MA, USA). The GAPDH proteins (1:5000; Abcam, Cambridge, MA, USA) were detected using an anti-rabbit HRP conjugated secondary antibody (1:10,000; Abcam, Cambridge, MA, USA). The immunoreactivity quantification was performed by means of densitometric analysis using Image J. The *MTHFR* protein levels were normalized to those of GAPDH.

### 2.6. Cell Invasion and Migration Assays

QGY-7703 and HL-7702 cells were grown to 60% confluence and were transfected with miR-NC, miR-22-3, and miR-149-5p mimic/inhibitor. A total of 36 h after transfection, the cells were seeded onto a Matrigel-coated membrane matrix (BD Biosciences, San Jose, CA, USA) in the insert of a 24-well culture plate (Costar, Corning, NY, USA). In the lower chamber, 500 μL of RPMI 1640 (QGY-7703) or DMEM (HepG2) with 10% fetal bovine serum was added as a chemoattractant. After 36 h, the non-invading cells were gently removed with a cotton swab. The invasive cells that were located on the lower surface of the chamber were stained with the 0.1% crystal violet and counted under a microscope in five predetermined fields (×200). The cell migration procedure is similar to the cell invasion procedure, with the exception of that the transwell membrane was not pre-coated with Matrigel. Either 18 (QGY-7703) or 24 h (HepG2) after transfection, the cell migration assay was performed. The cells that were adhered to the lower surface were in the counted the same way as they were during the cell invasion assay. All of the assays were repeated at least three times independently.

### 2.7. Statistical Analysis

The data were analyzed using the Statistical Package for Social Sciences version 21.0 (SPSS, Chicago, IL, USA). Student’s *t*-test for independent samples was used to compare the *MTHFR* protein, mRNA, or miRNA levels among the samples. The statistical hypothesis tests were two-sided, and a *p*-value < 0.05 was considered statistically significant.

## 3. Results

### 3.1. MiRNA-22-3p/miR-149-5p Are Downregulated in HCC Cell Lines

**Figure 1:**
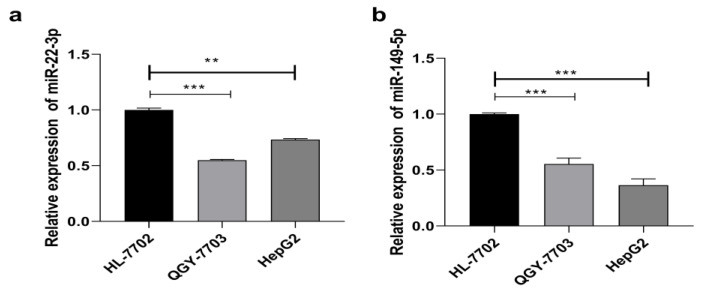
Expression of miR-22-3p in HCC cell lines and in normal liver cells. (**a**) Relative miR-22-3p level in HCC cells and in normal liver cells; (**b**) relative miR-149-5p level in HCC cells and in normal liver cells. (*n* = 3, ** *p* < 0.01, *** *p* < 0.001).

To evaluate the function of miR-22-3p/miR-149-5p in HCC, we first examined the endogenous transcriptional level of miR-22-3p/miR-149-5p in two human HCC cell lines (QGY-7703, HepG2) and one normal HL-7702 liver cells. Compared to normal liver cells, the transcription level of miR22-3p and miR-149-5p was significantly decreased in the HCC cell lines (Figure 1). 

### 3.2. MiR-22-3p/miR-149-5p Inhibit Hepatocellular Carcinoma Cells Proliferation

**Figure 2:**
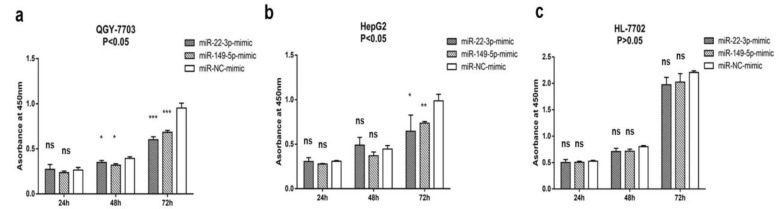
MiR-22-3p and miR-149-5p mimics suppress proliferation in human hepatocellular carcinoma cells but not in normal human hepatocyte cell. (**a**,**b**) Effect of miR-22-3p mimic (100 nM), miR-149-5p mimic (100 nM), and controlled miR-NC mimic (100 nM) on QGY-7703 and HepG2 cell proliferation was measured by the CCK8 assay at 24, 48, and 72 h post-transfection; (**c**) effect of miR-22-3p mimic (100 nM), miR-149-5p mimic (100 nM), and controlled miR-NC mimic (100 nM) on HL-7702 cell proliferation was measured by CCK8 assay at 24, 48, and 72 h post-transfection. Absorbance was read at 450 nm. Data are presented as mean ± S.D. (*n* = 3; * *p* < 0.05, ** *p* < 0.01, *** *p* < 0.001, ns stands for no significant difference).

We transfected miR-22-3p and miR-149-5p mimics into QGY-7703, HepG2, and HL-7702, and we then detected the changes in their proliferation levels. After the transfection of the miR-22-3p and miR-149-5p mimic in the QGY-7703 and HepG2 cells, the proliferation ability was significantly decreased after 24, 48, and 72 h, but no decrease was observed in HL-7702 at any time point. (Figure 2a–c).

### 3.3. MiR-22-3p/miR-149-5p Mimic Inhibit the Expression of MTHFR in HCC Cell Lines

**Figure 3:**
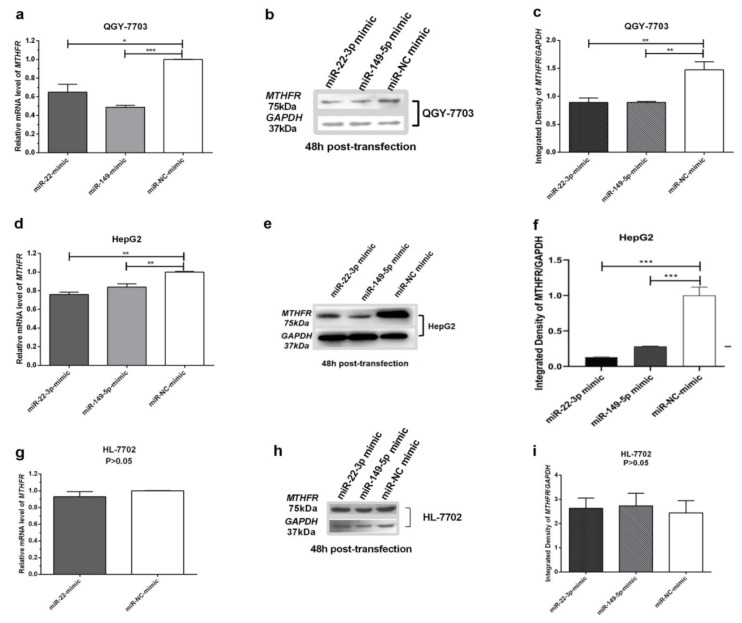
*MTHFR* mRNA expression and protein levels (after transfection for 48 h) in QGY-7703, HepG2, and HL-7702 cells after transfection with the miR-22-3p mimic (100 nM), miR-149-5p mimic (100 nM), and miR-NC mimic (100 nM); GAPDH served as an internal reference. (**a**) RT-qPCR assays revealed the mRNA expression of *MTHFR* in QGY-7703 cells after the transfection of miR-22-3p/miR-149-5p mimic; (**b**) Western blot assays showed the relative protein expression of *MTHFR* in QGY-7703 cells after transfection of miR-22-3p/miR-149-5p mimic; (**c**) densitometric analysis of protein bands in QGY-7703 cells after miR-22-3p/miR-149-5p mimic transfection; (**d**) RT-qPCR assays revealed the mRNA expression of *MTHFR* in HepG2 cells after miR-22-3p/miR-149-5p mimic transfection; (e) Western blot assays showed the relative protein expression of *MTHFR* in HepG2 cells after miR-22-3p/miR-149-5p mimic transfection; (**f**) densitometric analysis of protein bands in HepG2 cells after miR-22-3p/miR-149-5p mimic transfection; (**g**) RT-qPCR assays revealed the mRNA expression of *MTHFR* in HL-7702 cells after miR-22-3p mimic transfection; (**h**) Western blot assays showed the relative protein expression of *MTHFR* in HL-7702 cells after miR-22-3p/miR-149-5p mimic transfection. (**i**) Densitometric analysis of protein bands in HL-7702 cells after miR-22-3p/miR-149-5p mimic transfection; (*n* = 3; * *p* < 0.05, ** *p* < 0.01, *** *p* < 0.001).

Based on the results of previous studies, we analyzed the influence of the MiR-22-3p/miR-149-5p mimic on *MTHFR* expression. We found that when QGY-7703 and HL-7702 were transfected with the miR-22-3p/miR-149-5p mimic for 48 h, the *MTHFR* mRNA and protein levels were also significantly decreased in cell types (Figure 3a–f), but in the human normal hepatocyte HL-7702, no significant changes in the *MTHFR* mRNA and protein levels were observed compared to in the controls (Figure 3g–i).

### 3.4. Transfection of miR-22-3p/miR-149-5p Mimic Suppresses Migration and Invasion in HCC Cells In Vitro

**Figure 4:**
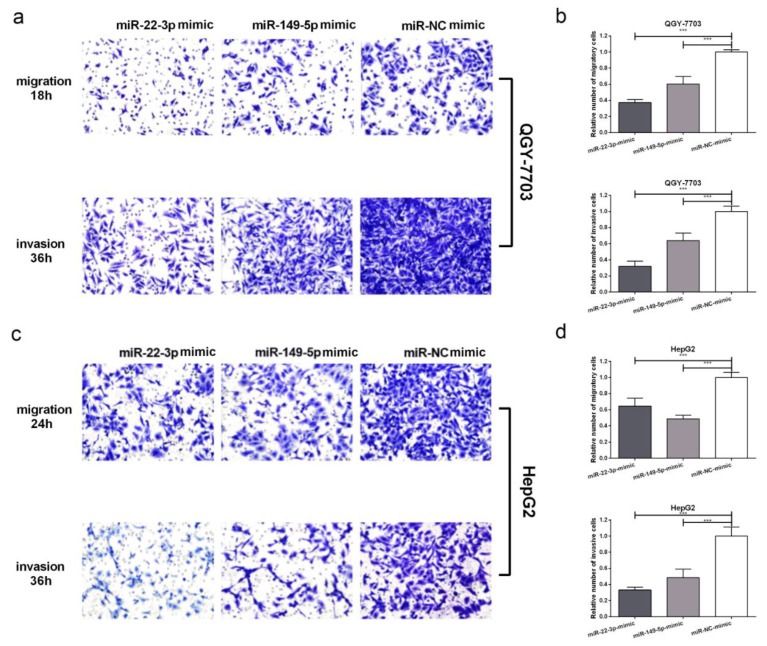
Representative images (**a**,**c**) and bar graphs (**b**,**d**) depicting the invasion and migration ability of QGY-7703 and HepG2 after miR-22-3p mimic (100 nM), miR-149-5p mimic (100 nM), or miR-NC mimic (100 nM) transfection. Data are presented as mean ± S.D. (*n* = 6) and analyzed by two-tailed unpaired *t*-test (×200 magnification, *** *p* < 0.001).

We then analyzed the effect of ectopic miR-22-3p/miR-149-5p expression on the cellular invasion and migration potential of QGY-7703 and HepG2 cells. During transwell invasion and the migration assay, the cells that had been transfected with miR-22-3p/miR-149-5p mimics displayed inhibition in terms of their invasion and migration ability when compared to the control group in the QGY-7703 (at 18 and 36 h) and HepG2 (at 24 and 36 h) cells (Figure 4a–d). Collectively, these results indicate that ectopic miR-22-3p/miR-149-5p significantly suppresses HCC cells proliferation, migration, and invasion in vitro.

### 3.5. Transfection of miR-22-3p/miR-149-5p Inhibitor Promote Migration in HCC Cells In Vitro

**Figure 5:**
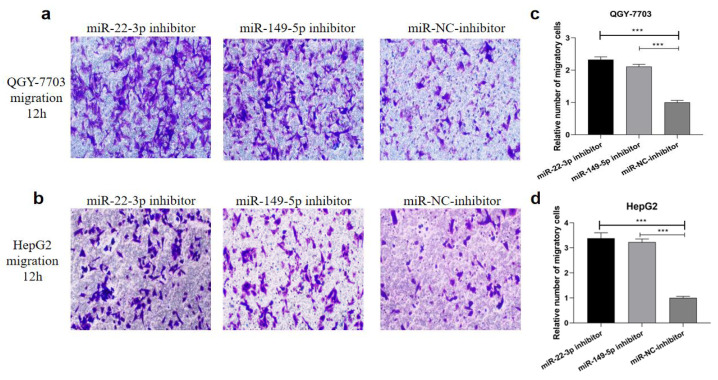
Representative images (**a**,**c**) and bar graphs (**b**,**d**) depicting the migration ability of QGY-7703 and HepG2 after miR-22-3p inhibitor (100 nM), miR-149-5p inhibitor (100 nM), or miR-NC (100 nM) transfection. Data are presented as mean ± S.D. (*n* = 6) and analyzed by a two-tailed unpaired *t*-test (×200 magnification, *** *p* < 0.001).

Subsequently, the effects of the miRNA-22-3p/miR-149-5p inhibitor on HCC cell migration were assessed. The transwell assay found that the miR-22-3p/miR-149-5p inhibitor significantly promoted HCC cell migration (Figure 5a–d and Figure S1).

**Figure 6:**
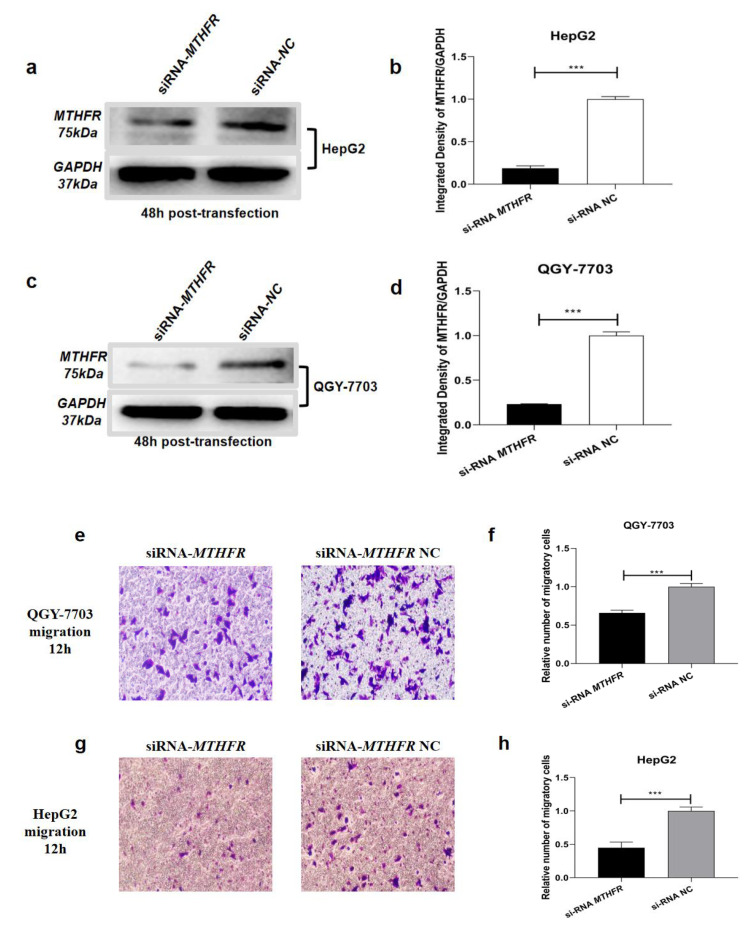
Western blot assays showed the relative protein expression of *MTHFR* in QGY-7703 and HepG2 cells after the transfection of ectopic expression of siRNA-MTHFR and siRNA-NC (**a**–**d**); representative images (**e**,**g**) and bar graphs (**f**,**h**) depicting the migration ability of QGY-7703 and HepG2 after siRNA-MTHFR (100 nM) and siRNA NC (100 nM). Data are presented as mean ± S.D. (*n* = 6) and analyzed by a two-tailed unpaired *t*-test (×200 magnification, *** *p* < 0.001).

To investigate whether *MTHFR* is necessary for miR-22-3p and miR-149-5p to inhibit HCC migration, we transfected si-RNA-*MTHFR* and si-RNA-*MTHFR* NC into HCC cells. Western blotting was performed to confirm the transfection efficiency (Figure 6a–d). Next, transwell assays were preformed to assess the influence of *MTHFR* on the motility of HCC cells in vitro, and the results indicated that siRNA-*MTHFR* is notably able to inhibit the HCC cell migration (Figure 6e–h). Based on the above results, it can be confirmed that miR-22-3p and miR-149-5p inhibit the migration and that the invasion properties of HCC may be partially able to be achieved through downregulating *MTHFR*.

### 3.6. Transfection of miR-22-3p/miR-149-5p Mimic Upregulation the Expression of PDCD4/TP53INP1 in QGY-7703

**Figure 7:**
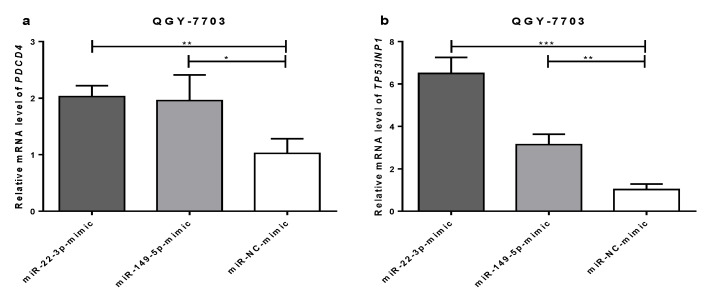
mRNA expression of *PDCD4* and *TP53INP1* in QGY-7703 after miR-22-3p mimic (100 nM), miR-149-5p mimic (100 nM), or miR-NC mimic (100 nM) transfection; GAPDH served as an internal reference. (**a**) RT-qPCR assays reveal the mRNA expression of *PDCD4* in QGY-7703 cells after transfection 48 h. (**b**) RT-qPCR assays reveal the mRNA expression of *TP53INP1* in QGY-7703 cells after transfection for 48 h. (*n* = 3; * *p* < 0.05, ** *p* < 0.01, *** *p* < 0.001).

To further explore the biological significance of the decreased *MTHFR* expression induced by miR-22-3p and miR-149-5p, we assessed the expression of the tumor suppressor genes *TP53INP1* and *PDCD4* in QGY-7703 that had been transfected with miR-22-3p/miR-149-5p mimic. The results showed that the transfection of the miR-22-3p mimic significantly increased TP53INP1 (6.50-fold) and PDCD4 (2.02-fold) expression (Figure 7a,b). Similarly, transfection with the miR-149-5p mimic increased *TP53INP1* (3.14-fold) and *PDCD4* (1.96-fold) expression in QGY-7703 (Figure 7a,b).

## 4. Discussion

MiR-22 and miR-149 has been found to be a regulator in diverse cancers, including in osteosarcoma, prostate cancer, cervical cancer, lung cancer [12], breast cancer [13], colorectal cancer [14,15], gastric cancer [16], and hepatocellular carcinoma [17], among others. A recent study showed that berberine treatment suppresses cancer cell growth by regulating miR-22 and SP1, which are downstream targets of CCND1 and BCL2 in HCC [18]. Chen et al. [19] reported that miR-22 exhibits tumor-suppressive effects in HCC cells by regulating YWHAZ/AKT/FOXO3a signaling. Luo et al. [20] showed that miR-22 was down-expressed in HCC and inhibited HCC cell proliferation, migration, and invasion through the downregulation of the cancer-associated gene CD147. Luo et al. [21] found that miR-149 is a potential prognostic biomarker for HCC and that the re-expression of PPM1F rescued the miR-149-mediated inhibition of cell migration and invasion. Our previous study [11] showed that these different regulatory mechanisms may depend on folate metabolism, and the present study demonstrates that ectopic miR-22 and miR-149 also significantly inhibit the HCC proliferation and metastasis as well as *MTHFR* expression in HCC.

Nutritional intervention, toxic exposure [22], and drugs may cause an increase in the expression levels of miR-22 and miR-149 in hepatocytes. In the study, miR-22-3p and miR-149-5p may inhibit the migration and invasion of HCC cells by regulating MTHFR. We found that folate deficiency can simultaneously increase the expression of miR-22 and miR-149, while miR-22 and miR-149 regulate MTHFR levels, and may act as an indirect methyl donor in the one carbon metabolism cycle and play an important role in maintaining methylation levels throughout the genome by regulating *MTHFR*, thereby regulating methylation level in the entire genome and genes. We found that miR-22-3p/miR-149-5p mimic transfection upregulated the expression of PDCD4/TP53INP1 in QGY-7703, and we speculate that the regulation of this methylation level is different in normal hepatocytes and in hepatoma cells, resulting in an inhibitory effect on the proliferation and metastasis of hepatoma cells but not in normal hepatocytes. At the same time, HCC cells migration was also notably inhibited after si-RNA-*MTHFR* transfection. Therefore, this study demonstrates that ectopic miR-22 and miR-149 can also significantly inhibit the proliferation and metastasis of hepatoma cells by downregulating *MTHFR* expression. Other studies have also found that miRNAs can affect the proliferation and migration of cancer cells by regulating the expression of TP53INP1 and PDCD4 [23,24].

We also found that the inhibitors of miR-22-3p or miR-149-5p in both of the transfected cell lines and that the *MTHFR* expression was not restored in the HCC cell line (data not show here). We supposed that *MTHFR* had a long 3′UTR (4957bp) structure with more than one miRNA, such as miR-22, miR-149, miR-214, etc., which directly targeted the *MTHFR* 3′UTR and that many other potential miRNAs (predicted by TargetScan and miRDB) might also target the *MTHFR* 3′UTR. Therefore, we supposed that the deletion effect caused by one or two miRNAs might be limited, and complex regulation mechanisms need to be further investigated. In total, we think that the decreased expression of *MTHFR* expression caused by the miR-22-3p and miR-149-5p mimic might inhibit the promoter methylation of *TP53INP1* and *PDCD4*, thereby increasing TP53INP1 and PDCD4 expression and tumor suppression.

## 5. Conclusions

In summary, ectopic miR-22-3p/miR-149-5p inhibits the proliferation, migration, and invasion hepatocellular carcinoma cells but do not inhibit normal human hepatocytes proliferation. The expression of *MTHFR* were inhibited in two hepatocellular carcinomas but not in normal hepatocytes after the transfection of the ectopic miR-22-3p/miR-149-5p. This study suggests that miRNA-22-3p and miRNA-149-5p inhibit tumor growth and metastasis properties in human hepatocellular carcinoma may be partially by regulating the methylenetetrahydrofolate reductase. Ectopic miR-22-3p/miR-149-5p exerts anticancer effects directly but does not affect the proliferation of normal human hepatocytes. Therefore, it could be used as a potential target for the treatment of hepatocellular carcinoma.

## Data Availability

The data used and analyzed in this study will be made promptly available upon request.

## Supplementary Materials

The following supporting information can be downloaded at: https://www.mdpi.com/article/10.3390/cimb44020063/s1, Figure S1 Additional representative images (a) and bar graphs (b) depicting the migration ability of HepG2 after miR-22-3p inhibitor (100 nM), miR-149-5p inhibitor (100 nM) or miR-NC (100 nM) transfection. Additional representative images (c) and bar graphs (d) depicting the migration ability of QGY-7703 after siRNA-MTHFR (100 nM), siRNA-MTHFR NC (100 nM). Data are presented as mean ± S.D. (n = 6) and analyzed by a two-tailed unpaired *t*-test (×200 magnification, *** *p* < 0.001).
