# Correction: Chao et al. MicroRNA-22-3p and MicroRNA-149-5p Inhibit Human Hepatocellular Carcinoma Cell Growth and Metastasis Properties by Regulating Methylenetetrahydrofolate Reductase. *Curr. Issues Mol. Biol.* 2022, *44*, 952–962

**DOI:** 10.3390/cimb46090630

**Published:** 2024-09-23

**Authors:** Chao Li, Xiang Li, Han Wang, Xihan Guo, Jinglun Xue, Xu Wang, Juan Ni

**Affiliations:** 1 School of Life Sciences, The Engineering Research Center of Sustainable Development and Utilization of Biomass Energy, Yunnan Normal University, Kunming 650500, China; lichao@fudan.edu.cn (C.L.); lixiang7368@163.com (X.L.); wanghan9215@163.com (H.W.); guoxh1987@163.com (X.G.); 2 State Key Laboratory of Genetic Engineering, Institute of Genetics, School of Life Sciences, Fudan University, Shanghai 200438, China; jlxue@fudan.edu.cn

Author Correction: We apologize for unintentionally using the wrong figures (Figure 5b and Figure 6e) in the original article. To correct our mistakes, representative images (Figure 5b) and bar graphs (Figure 5d) depicting the migration ability of HepG2 after miR-149-5p inhibitor (100 nM) transfection are provided, and the legends have also been modified accordingly. Representative images (Figure 6e) and bar graphs (Figure 6f) depicting the migration ability of QGY-7703 after siRNA-*MTHFR* (100 nM) and siRNA-*MTHFR* NC (100 nM) transfection are also provided. In order to ensure the reliability of the experimental results, we repeated the experiment six times for the new pictures, and this part of the results was included in the Supplementary Materials (Figure S1).

The corrected Figure 5b,d and Figure 6e,f appear below:


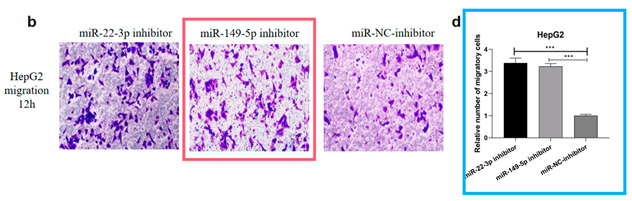



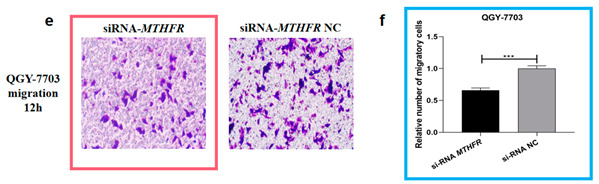


In the original publication, the Supplementary Materials was not included. The citation has now been inserted in Section 3.5 Transfection of miR-22-3p/miR-149-5p Inhibitor Promote Migration in HCC Cells In Vitro, Paragraph 1 and should read:

Subsequently, the effects of the miRNA-22-3p/miR-149-5p inhibitor on HCC cell migration were assessed. The transwell assay found that the miR-22-3p/miR-149-5p inhibitor significantly promoted HCC cell migration (Figures 5a–d and S1).

The corrected Supplementary Materials statement appears here. The authors state that the scientific conclusions are unaffected. This correction was approved by the Academic Editor. The original publication has also been updated.

## Supplementary Materials

The following supporting information can be downloaded at: https://www.mdpi.com/article/10.3390/cimb44020063/s1, Figure S1 Additional representative images (a) and bar graphs (b) depicting the migration ability of HepG2 after miR-22-3p inhibitor (100 nM), miR-149-5p inhibitor (100 nM) or miR-NC (100 nM) transfection. Additional representative images (c) and bar graphs (d) depicting the migration ability of QGY-7703 after siRNA-MTHFR (100 nM), siRNA-MTHFR NC (100 nM). Data are presented as mean ± S.D. (n = 6) and analyzed by a two-tailed unpaired *t*-test (×200 magnification, *** *p* < 0.001).
