# Supplementary material for: MicroRNA-22-3p and MicroRNA-149-5p Inhibit Human Hepatocellular Carcinoma Cell Growth and Metastasis Properties by Regulating Methylenetetrahydrofolate Reductase
Source: Curr Issues Mol Biol. 2022 Feb 16;44(2):0. doi: 10.3390/cimb44020063 (PMC8928992; doi:10.3390/cimb44020063)
Supplement: Supplementary file 1 [file cimb-44-00063-s001.zip › cimb-1559462-Supplementary.pdf]

Supplementary information，Figure S1

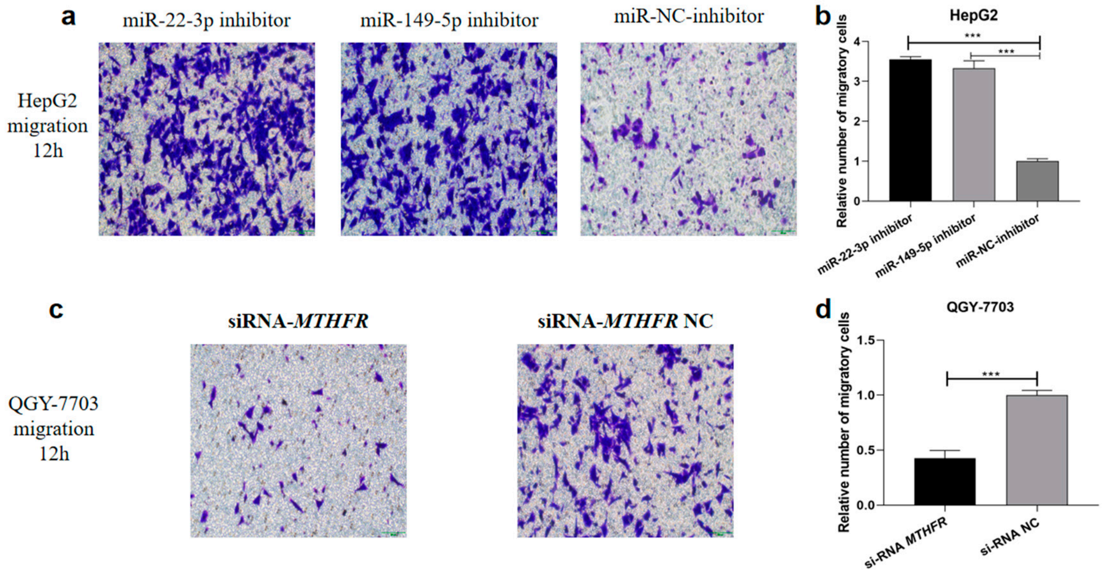

Figure S1 Additional representative images (a) and bar graphs (b) depicting the migration ability of HepG2 after miR-22-3p inhibitor (100 nM), miR-149-5p inhibitor (100 nM) or miR-NC (100 nM) transfection. Additional representative images (c) and bar graphs (d) depicting the migration ability of QGY-7703 after siRNA-*MTHFR* (100 nM), siRNA-*MTHFR* NC (100 nM). Data are presented as mean ± S.D. (n=6) and analyzed by a two-tailed unpaired t-test (×200 magnification, ***P<0.001).
